# Ureteral Stricture after Laparoscopic Tubal Ligation due to Suturing of the Serosa 

**DOI:** 10.1155/2012/546989

**Published:** 2012-10-30

**Authors:** Berat Cem Özgür, Ahmet Metin Hasçiçek, Tolga Karakan, Cem Nedim Yücetürk, Erim Ersoy

**Affiliations:** Ankara Research and Training Hospital, Ankara, Turkey

## Abstract

Strictures secondary to traumas of the ureter are some of the complications of urogynecologic surgery. We present a 43-year-old female who had a history of laparoscopic tubal ligation a year ago and was admitted to our department with recurrent flank and inguinal pain. It was soon understood that a suture has pulled the ureter from the lateral serosa of the upper part to the lateral serosa of the lower part causing dilatation of the proximal and midureter because of the previous surgery while there was no damage on the ureteral lumen. Consequently successful reconstruction was performed with open ureteroureterostomy.

## 1. Introduction

Ureteral injuries and secondary strictures are known complications of urogynecologic surgery. Ureteral stenting and endoscopic balloon dilation are effective techniques that can be used for the management of ureteral strictures after urogynecologic surgery complications and avoids further surgical intervention. In cases in which such techniques for the management of distal obstruction were unsuccessful ureteral reimplantation with laparotomy and recently laparoscopy have been used. We present a case of a 43-year-old female who had a history of laparoscopic tubal ligation a year ago and kinking of the left ureter was noted after the surgery.

## 2. Case

**Figure 1:**
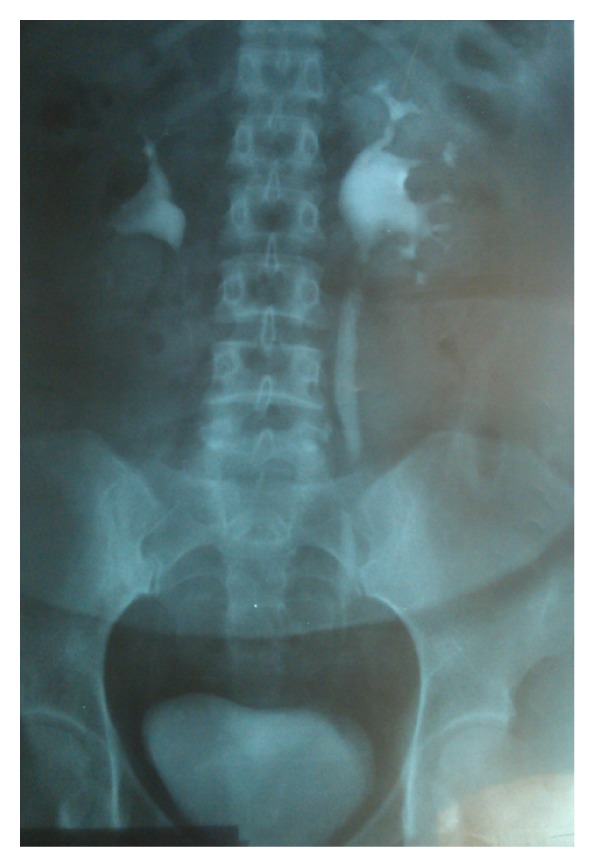
Excretory urography showed dilatation of left ureter.

A 43-year-old female previously had a laparoscopic tubal ligation, presented with recurrent left flank and inguinal pain. The pain was severe in nature. She had similar episode three months prior to presentation. The symptoms at that time were relieved with analgesics, antibiotics, and infusions. Also three urinary tract infections occurred in the previous year after the laparoscopic tubal ligation and treated with antibiotherapy. Pulse rate of 90/min, a respiratory rate of 34 cycles/min, and a blood pressure of 110/90 mmHg were detected. Abdominal examination revealed tenderness in the left lumber and left hypochondrial regions. No masses were palpable within the abdomen. The cardiovascular, neurologic examinations were essentially normal. Ultrasonography revealed Grade 2 hydronephrosis of the left kidney. She was referred to our center for further management, after an unsuccessful attempt at retrograde balloon dilatation and ureteral stent insertion to the left ureter because of hydronephrosis. Both an IVU (intravenous urogram) and retrograde ureterogram were performed to determine the site and degree of stricture at that center (Figure 1). 

**Figure 2:**
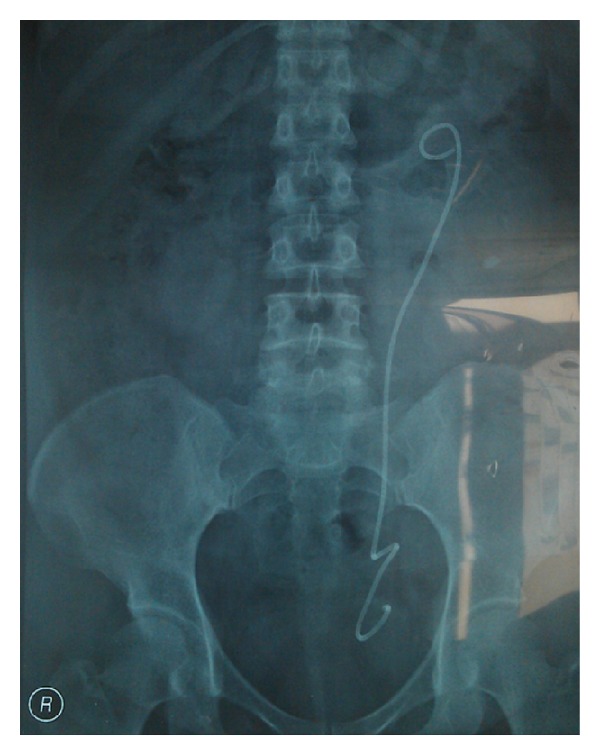
The Omega-like shape of the lower ureter after double J cathether insertion.

**Figure 3:**
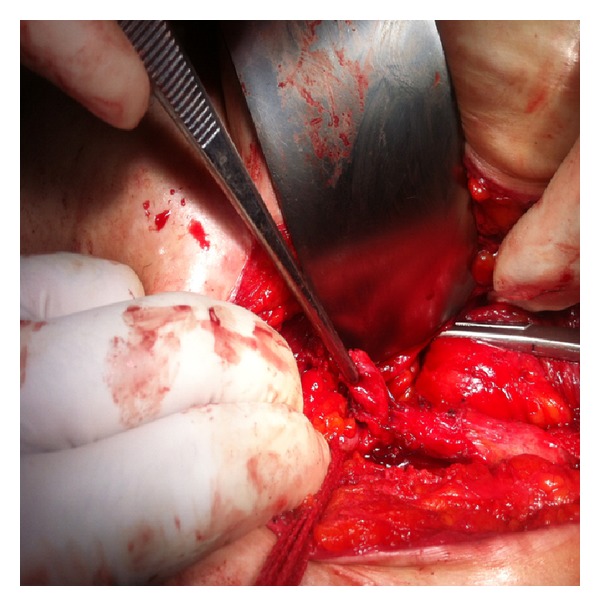
Intraoperatively a kinking of the ureter like an Omega shape because of the suturing is seen.

 At our hospital ureteroscopy guided double J stenting was planned initially. A 4.8 fr double J stent was inserted with the help of fluoroscopy as shown in Figure 2. After 2 months followup, regression of the hydronephrosis was seen on ultrasonography. But the pain persisted in going on. After discussing benefits and the risks of various treatment modalities, open surgical exploration was planned. The exploration was done via a left Gibson incision. Dilatation of the proximal and midureter and an area of stricture was observed in distal ureter nearly 5 cm in length. Interestingly the shape of the ureter was like an Omega (*ω*) at that region and as the exploration was done, it was understood that a suture has pulled the ureter from the lateral serosa of the upper part to the lateral serosa of the lower part as seen in Figure 3. Also the luminal segment of the ureter was intact so that a double j stent could be inserted. Intraoperatively the strictured area was excised and a successful reconstruction was performed with open ureteroureterostomy. The patient remains asymptomatic, with normal renal sonogram, 2 months after the procedure.

## 3. Discussion 

 The ureter is injured in 1-2% of routine gynecological pelvic operations [1]. The injury may heal spontaneously or may be accompanied by an iatrogenic fistula in up to 10% of cases [2]. Another complication of those injuries are strictures that may obstruct the upper urinary system. Lower ureteric strictures need immediate attention to ensure unobstructed drainage of urine from the renal unit. In iatrogenic lower ureteric injuries conventional practice entails attempting a ureteral stent or nephrostomy primarily, deferring definitive reconstruction for an interval [3]. Definitive reconstruction differs from the endoscopic procedures to open and laparascopic approaches due to many factors like the time of diagnosis, location and the length of the injury, the presence of surgical or medical illness, the experience of the surgeon, and so forth [4]. Treatment has progressively shifted from ureteroneocystostomy or ureteroureterostomy performed by laparotomy to less invasive treatment options such as ureteral stenting or dilatation in case of stricture, stenting under laparoscopic guidance and laparoscopic stitching of lacerations, laparoscopic ureteral reanastomosis, or laparoscopic ureteroneocystostomy and ureteroureterostomy [5].

 Partial and segmental stenoses can be treated by endoscopic procedures such as dilation or internal ureterotomy with placement of double J catheter with good follow-up results. Consideration of its minimal invasiveness and acceptable long-term outcome, simple retrograde balloon dilation is an effective treatment modality for benign ureteral stricture with a short segment (≤2 cm), and a shorter duration of stenting (3-weeks) is viable. The success of that procedure rate varies from 20% to 85% [6–8]. 

 In our patient the segment of the ureter was nearly 5 cm long in preoperative images and we planned open surgery (ureteroureterostomy). The main reason for choosing such technique was our clinical experience as the laparoscopic reconstruction might be the first option in experienced centers [9]. Reconstruction technique procedures are needed for total complex stenosis and the ideal time to perform this reconstruction remains controversial. Some authors recommend a minimum time of 6 weeks after the injury prior to carrying out a new surgical operation in cases of lesions caused by surgical trauma, in order to allow maximum resolution of the inflammatory process. In our case there was no need to think about the optional time for surgery since the previous gynecological surgery was nearly a year ago. There has been a long time from the laparoscopic tube ligation to the symptoms occurred and in our opinion, it was because the suturing had only passed through the serosal part of the ureter and the luminal part was intact so that a 4.8 fr DJ catheter was easily inserted in the first procedure. The main problem was a serosal suturing that brought closer the upper and lower lateral serosal parts of the ureter and the kinking occured in omega shape. Only cutting the lateral suturing might solve that problem but there was an intense fibrotic reaction of the surrounding tissue and the ureter was long enough for an easy reconstruction so that the fibrotic segment was excised and ureteroureterostomy was performed.

Gynecologic surgery accounts for more than 50 percent of all ureteral injuries resulting from an operation, with the remaining occurring during colorectal, general, vascular, and urologic surgery [10, 11]. Awareness of ureter protection should be enhanced within laparoscopic surgery. Although most operative complications occurred in advanced operative procedures and despite the advanced technology and experience, complications during the installation phase of laparoscopy remain a cause of different morbidities also in easier surgeries like tubal ligation.

 An inflammatory reaction or trauma that does not affect the ureteral lumen must be kept in mind in case of long-lasting pain recurrent urinary tract infections. Stricture develops when a ureter with deficient blood supply, often from a certain type dissection, heals by scar tissue. Side or abdominal pain and urinary tract infection/pyelonephritis are commonly seen. Ureteral strictures that are diagnosed within 6 to 12 weeks, and are relatively short in length, can be managed successfully by balloon dilatation or endoscopic incision and stenting [12]. For endoscopic failures, when the stricture is discovered late, in particularly dense or long, or radiation induced, an open or laparoscopic surgical repair is necessary.
